# Framework to guide the use of mathematical modelling in evidence-based policy decision-making

**DOI:** 10.1136/bmjopen-2024-093645

**Published:** 2025-04-05

**Authors:** Jacquie Oliwa, Fatuma Hassan Guleid, Collins J Owek, Justinah Maluni, Juliet Jepkosgei, Jacinta Nzinga, Vincent O Were, So Yoon Sim, Abel W Walekhwa, Hannah Clapham, Saudamini Dabak, Sarin KC, Liza Hadley, Eduardo Undurraga, Brittany L Hagedorn, Raymond Cw Hutubessy

**Affiliations:** 1 Health Services Unit, KEMRI-Wellcome Trust Research Programme, Nairobi, Kenya; 2 Public Health, Institute of Tropical Medicine, Antwerp, Flanders, Belgium; 3 Health Economics Research Unit, KEMRI-Wellcome Trust Research Programme Nairobi, Nairobi, Kenya; 4 Department of Public and Global Health, University of Nairobi, Nairobi, Kenya; 5 Data Synergy and Evaluation Unit, African Population and Health Research Center, Nairobi, Kenya; 6 World Health Organization, Geneva, Switzerland; 7 Diseases Dynamics Unit, Department of Veterinary Medicine, University of Cambridge, Cambridge, UK; 8 National University of Singapore, Singapore; 9 Health Intervention and Technology Assessment Program, Muang, Nonthaburi, Thailand; 10 Disease Dynamics Unit, University of Cambridge, Cambridge, UK; 11 London School of Hygiene & Tropical Medicine, London, UK; 12 Pontificia Universidad Catolica de Chile, Santiago, Chile; 13 Bill & Melinda Gates Foundation, Seattle, Washington, USA

**Keywords:** COVID-19, Health policy, Decision Making

## Abstract

**Abstract:**

**Introduction:**

The COVID-19 pandemic highlighted the significance of mathematical modelling in decision-making and the limited capacity in many low-income and middle-income countries (LMICs). Thus, we studied how modelling supported policy decision-making processes in LMICs during the pandemic (details in a separate paper).

We found that strong researcher–policymaker relationships and co-creation facilitated knowledge translation, while scepticism, political pressures and demand for quick outputs were barriers. We also noted that routine use of modelled evidence for decision-making requires sustained funding, capacity building for policy-facing modelling, robust data infrastructure and dedicated knowledge translation mechanisms.

These lessons helped us co-create a framework and policy roadmap for improving the routine use of modelling evidence in public health decision-making. This communication paper describes the framework components and provides an implementation approach and evidence for the recommendations. The components include (1) funding, (2) capacity building, (3) data infrastructure, (4) knowledge translation platforms and (5) a culture of evidence use.

**Key arguments:**

Our framework integrates the supply (modellers) and demand (policymakers) sides and contextual factors that enable change. It is designed to be generic and disease-agnostic for any policy decision-making that modelling could support. It is not a decision-making tool but a guiding framework to help build capacity for evidence-based policy decision-making. The target audience is modellers and policymakers, but it could include other partners and implementers in public health decision-making.

**Conclusion:**

The framework was created through engagements with policymakers and researchers and reflects their real-life experiences during the COVID-19 pandemic. Its purpose is to guide stakeholders, especially in lower-resourced settings, in building modelling capacity, prioritising efforts and creating an enabling environment for using models as part of the evidence base to inform public health decision-making. To validate its robustness and impact, further work is needed to implement and evaluate this framework in diverse settings.

**Summary**

- The COVID-19 pandemic led more decision-makers and researchers to see the practical application and significance of mathematical modelling in decision-making.
- The pandemic further highlighted the limited capacity for policy-facing modelling in many low-income and middle-income countries.
- We developed a framework and policy roadmap for improving the routine use of modelling evidence in public health decision-making in lower-resource settings.
- The overall goal of the framework is to enable routine use of reliable, timely and locally generated mathematical modelling evidence to inform public health decisions for better health outcomes.
- The key components include (1) sustainable funding, (2) capacity building for the generation of local modelling estimates, (3) the availability of robust data infrastructure, (4) knowledge translation and (5) a culture of evidence use.
- Further work is needed to implement and evaluate this framework in diverse settings.

## Introduction

 Public health emergencies such as disease outbreaks disrupt populations and may follow uncertain trajectories, particularly in resource-limited settings with poor surveillance.^1^,^3^ Outbreaks require prompt and effective responses guided by the best available evidence to minimise harm and reduce the risk of spread.^4^,^6^ The COVID-19 pandemic presented a unique challenge and an opportunity to learn about using evidence for decision-making during a rapidly evolving emergency.^7 8^ While many sources of evidence were used to guide COVID-19 responses, mathematical modelling was at the forefront.^9^,^11^

When COVID-19 emerged, little was known about how many people would become infected, how far it could spread, the effectiveness of public health interventions or how resources should be optimally allocated.^12 13^ The unprecedented urgency to make decisions to slow down transmission (‘flatten the curve’) and minimise harm meant that timely evidence was critical.^14^ Direct experimentation and observation of the novel virus would be unethical and impractical. Considering this, policymakers globally turned to researchers for answers. Mathematical modelling provided much-needed evidence to guide policy decisions throughout the pandemic.^15^,^17^ However, despite its relevance, policy-facing modelling (ie, creating and using mathematical models tailored to inform policy decisions in public health) was not extensively used in many low-resource settings.^18^

### The usefulness of mathematical modelling in informing public health policy

Mathematical modelling is a scientific approach to explaining an observed phenomenon and testing this formulation to demonstrate the outcome of various experiments under various conditions.^19^ Models provide a versatile platform for studying transmission, progression and mitigation scenarios, exploration of future trends and forecasting potential outcomes, for example, during an outbreak.^20 21^ They may help characterise the affected communities’ epidemiological status (state of the population in a specific time and place about a particular disease or health condition), the infectious agent’s transmissibility and the potential impact of public health interventions.^9^ This is important when biological processes may not be fully understood, yet decisions must be made quickly. Furthermore, models can identify risk factors to inform targeted disease control and prevention strategies.^20^ By engaging in collaborative modelling, researchers and policymakers can develop a shared understanding of an uncertain future and how to navigate it effectively, using mathematical equations to replicate real-life scenarios and predict likely outcomes with and without interventions.^22 23^ Mathematical modelling thus provides an invaluable tool for making explicit assumptions, highlighting key factors determining policy needs and providing quantitative predictions for the effectiveness and cost-effectiveness of policies.^19^

Mathematical models have historically been instrumental in informing policy decisions in emergency and non-emergency settings. For instance, the influenza modelling community aided outbreak response planning during the 2009 pandemic by characterising the dynamics and impact of the H1N1 virus.^24 25^ Models also projected outcomes for endemic diseases like tuberculosis and epidemics such as the 2014–2015 Ebola outbreak in West Africa.^26 27^ Additionally, models showed that universal voluntary HIV testing and immediate antiretroviral therapy could significantly reduce future HIV transmission.^28^ Mathematical models have also been used as part of the evidence review process by the WHO Strategic Advisory Group of Experts on Immunization (SAGE).^29^

Before the pandemic, several countries (mainly high-income) routinely used mathematical modelling to guide policy decisions. For instance, in the UK, the Scientific Pandemic Influenza Group on Modelling^30^ and other academic institutions closely collaborate with the Department of Health and SAGE.^31 32^ The Institute Pasteur in France, the Robert Koch Institute in Germany and the National Institute of Public Health and the Environment in the Netherlands all work with dedicated policy-facing modelling teams.^33^ In North America, the US Centers for Disease Control and Prevention and the Public Health Agency of Canada work with internal modelling groups and actively collaborate with modellers in academia.^33^

The COVID-19 pandemic underscored the practical importance of mathematical modelling for decision-making worldwide while highlighting the limited capacity for policy-facing modelling in many low-income and middle-income countries (LMICs).^34 35^ There were, however, some notable exceptions; for instance, in Nigeria, policymakers collaborated closely in real time with mathematical modellers and epidemiologists, in a co-production multidisciplinary process to inform decision-making.^36^ Likewise, the Eastern Mediterranean Region used a participatory approach, engaging decision-makers and public health professionals in using model outputs to inform policy decisions for pandemic control.^37^ Nevertheless, in many LMICs, the role and extent of modelling in decision-making remain unclear, with limited studies assessing the use of modelling in public health practice.^33 38 39^

This communication paper describes the process we used to develop a framework to help build capacity for evidence-based decision-making, using information from literature and our study described elsewhere.^40 41^

The study investigated the use of mathematical modelling to inform policy decisions during the COVID-19 pandemic, focusing on lower-resource settings. Data for the mixed-methods study were obtained from a survey, a scoping review, in-depth interviews and participant observer notes from learning workshops with researchers and policy actors mainly from Africa, Southeast Asia and Latin America. While acknowledging the substantial heterogeneities in data governance, quality, decision-making processes and capabilities among LMIC countries, we aimed to identify common themes, to understand how modelling data were used for decision-making during the pandemic, the challenges faced and the necessary actions and resources needed to be in place for future emergencies and non-pandemic periods.

**Figure 1:**
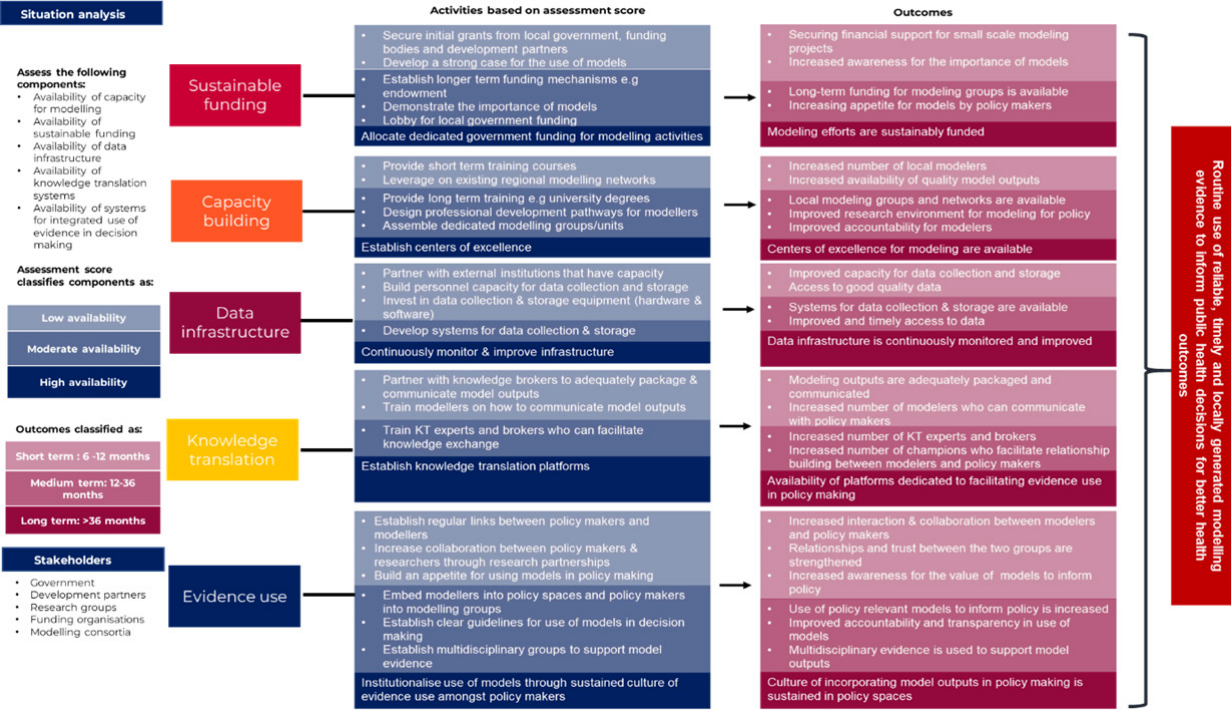
Framework to improve the use of modelling evidence to guide policy decision-making.^41^

We found that effective use of modelled evidence requires capacity building for policy-facing modelling, robust data infrastructure, sustained funding and dedicated knowledge translation mechanisms. Strong researcher–policymaker relationships and co-creation facilitated knowledge translation, while scepticism, political pressures and demand for quick outputs posed as barriers. The lessons learnt helped us develop a framework (figure 1) for improving the routine use of modelling evidence in public health policymaking in lower-resource settings, where this was probably not the norm before the pandemic.^41 42^ The framework was developed using a theory of change approach, where participants reflected on data from the study and came up with a priority of strategies to increase knowledge translation efforts systematically. The framework’s purpose is thus to guide stakeholders in lower-resourced settings in building modelling capacity, prioritising efforts and creating an enabling environment for using models as part of the evidence base that informs public health decision-making. The framework is designed to be generic and disease-agnostic for any policy decision-making that modelling could support.

## Framework to improve the use of modelling evidence to guide policy decision-making

The overall goal of the framework is to enable routine use of reliable, timely and locally generated mathematical modelling evidence to inform public health decisions for better health outcomes. The target audience is modellers and policymakers but could include other partners and implementers in public health decision-making. Given the diverse contextual factors across the different decision-making spaces, the framework does not propose one single approach for incorporating modelling evidence in decision-making. Instead, it highlights five interdependent components that should be considered to achieve this goal, as illustrated in figure 1^41^ and in the simplified road map for policymakers in online supplemental file S1.

The framework should be adapted to existing local capacity, which requires a situational analysis to assess the availability and adequacy of the various components and identify existing needs and challenges. The framework outlines specific activities for each element contingent on availability (shaded blue in figure 1) with desired outcomes for each activity (shaded red). The outcomes can be used as a guide to monitor progress. This adaptable approach ensures that the framework can be implemented in diverse settings to promote the integration of mathematical models into routine decision-making processes.

### Components of the framework

The framework components integrate the supply (modellers) and demand (policymakers) sides and contextual factors that enable change. The five interdependent components are (1) sustainable funding, (2) capacity building for the generation of locally relevant modelling estimates, (3) the availability and access to robust data infrastructure, (4) knowledge translation and (5) a climate of evidence use, and we tackle each component in detail in the subsequent sections.

#### Sustainable funding

Unlike many scientific disciplines, modelling does not require substantial investment in equipment, physical laboratories and consumables. The primary investments mainly involve building modelling capacity by training and retaining modellers and enabling robust, high-quality, accessible data infrastructure. Work by Results for Development (R4D) showed that in most countries funding for modelling is mostly from bilateral agencies and international organisations such as the WHO, the World Bank and Bill & Melinda Gates Foundation, with some support from local governments.^43^ Heavy reliance on international organisations can be an obstacle to the sustainability of policy modelling, with attendant concerns for lack of autonomy. Reliance on external funding may result in modelling efforts more closely aligned with funders’ priorities rather than local priorities. Modelling expertise is often undervalued within LMIC public health institutions, which may contribute to insufficient investment by governments.^33^

Recognising the critical role of modellers requires adequately remunerating them, fostering motivation and encouraging their contributions to generate evidence for policy decisions. The COVID-19 pandemic underscored this need, with several researchers from LMICs reporting facing challenging conditions while working under pressure, sometimes without salaries, and often facing job insecurity.^44^

**Figure 2:**
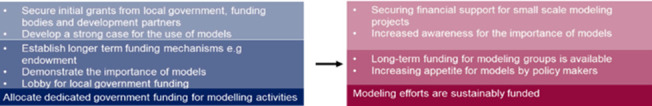
Strategies for sustainable funding and desired outcomes.^41^

We suggest that a pathway towards sustainable funding for countries with little to no funding to build an effective modelling ecosystem could involve initiating short-term grants from funding bodies and development partners, as shown in figure 2.^41^ These short-term grants/seed funds can be used for small projects to demonstrate the utility of models. Successful outcomes could then be leveraged to attract larger amounts of funding. As the case for modelling becomes more compelling and local capacity increases, modelling groups can transition from project-based funding to stable, longer-term ‘core’ funding to support their research activities. Sustained funding can take various forms, such as endowment funds, line-item budgeting and government grants. The desired outcomes include increased public health decision-makers’ awareness of the significance of models in supporting decision-making, coupled with growing demand from policy actors requiring long-term sustainable funding to support modelling efforts, as shown in figure 2 from the framework.^41^

#### Capacity building for policy modelling

Alongside funding, effective policy modelling requires a cadre of local researchers with sufficient data wrangling and modelling expertise. Policymakers, in turn, need the ability to appraise research quality and collaborate with other stakeholders to translate evidence into actionable policies. Local researchers and institutions are better positioned to understand the context and needs of their communities, available data strengths and limitations, and better interpret model outcomes and implications.^44^ Neglecting contextual factors in modelling efforts has been shown to produce less useful projections.^45^ The COVID-19 pandemic highlighted the insufficient local capacity to conduct policy-facing modelling in many LMICs, resulting in reliance on external modellers, who may have lacked a good understanding of the local context.^34^

Building local capacity for mathematical modelling involves training in the methods and providing resources and infrastructure for research. They need access to reliable data, literature and interdisciplinary teams (eg, doctors, entomologists, veterinarians, behavioural scientists) to interpret data collaboratively. To generate outputs that effectively inform policy decisions, modellers also require expertise and training in contextualising their findings and financing and investment case development.^34^ Finally, they will need capacity building in knowledge translation and communication, among other soft skills, to navigate political complexities and engage meaningfully with policymakers.^35^

**Figure 3:**
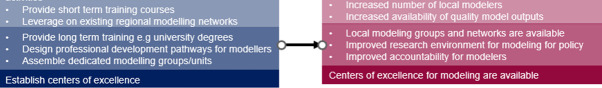
Strategies for capacity building and desired outcomes.^41^

As seen from the framework in figure 3,^41^ a practical pathway to increasing capacity in policy-facing modelling is education and training, initially through short courses, workshops, journal club sessions within regional networks and, in the longer term, establishing formal academic programmes at universities, with mentorship on completion. These programmes should provide a comprehensive understanding of the concepts and methods while highlighting the importance of policy-relevant modelling. Beyond acquiring technical expertise, achieving policy impact requires collaboration with decision-makers, active engagement and effective knowledge translation.

In addition, capacity can be built through hands-on experience in joint research projects, working with experienced modellers and epidemiologists from other countries. Such collaborations offer access to valuable expertise, technical resources and technological infrastructure. As countries continue to cultivate a pool of local modellers, building and strengthening national and regional networks of modellers becomes essential. These networks can serve as platforms for sharing knowledge, resources and expertise. They can also help coordinate research efforts, foster collaboration, improve accountability and enhance the quality of model estimates while contributing to informed policy decisions.

Over time, the networks can develop into centres of excellence (CoE) dedicated to modelling to help inform policy decision-making. CoEs can be central in providing leadership, nurturing capacity, defining best practices and supporting modelling for public health policy. This is critical for facilitating model outputs to inform public health decisions, particularly in health emergencies. Furthermore, as modelling is just one source of evidence, CoEs can incorporate other disciplines, such as public health, epidemiology, health economics and behavioural sciences, to ensure that findings from models are integrated within the broader evidence ecosystem, fostering a comprehensive approach with interdisciplinary and multisectoral collaboration.

The anticipated outcomes of these strategies include an increased pool of local modellers producing context-specific, policy-relevant models in the short term, local modelling groups in a research environment for policy-facing modelling in the medium term and established CoEs in the long term, as shown in figure 3. These CoEs, driven by local expertise and experience and integrated into regional networks, will likely ensure trust from decision-makers. Their sustained presence would significantly improve evidence-based decisions in public health.

There might be an apparent tension between the need for locally relevant modelling and the importance of establishing broader collaboration networks. The COVID-19 pandemic demonstrated the interconnectedness of the global community and the necessity of international cooperation in addressing public health emergencies.^37 45^ While local modelling is essential for informing contextual policy decision-making, fostering wider collaborative networks is equally crucial to facilitating the exchange of expertise and resources. This allows researchers and policymakers to glean insights from each other’s experiences and collectively devise more effective strategies. Therefore, balancing local relevance and global collaboration can foster a more effective and adaptable approach to modelling in public health decision-making.

An excellent example of a network approach that supported LMICs during the pandemic is the COVID-19 Modelling (CoMo) Consortium, a network of modellers and other public health experts from over 40 countries in Africa, Asia and the Americas.^45^ The CoMo Consortium uses a participatory approach to provide policymakers with decision-making support using epidemiological and economic models adapted to each country’s context. Experts from country teams led modelling during the pandemic, working closely with their counterpart policymakers. At the same time, researchers from the UK and the USA provided technical and consultative support.

Another example of regional network CoE is the Pan American Health Organization’s Provac Initiative, where the CoEs established led to the development and piloting of tools, methodological guides, and training materials to support countries in generating new evidence for vaccine introduction.^46^

#### Data infrastructure

High-quality, readily available and accessible data were needed to understand the COVID-19 pandemic and public health interventions. This included data on case counts, viral transmissibility, population mobility, policy implementation, clinical symptoms, hospitalisations, treatments, diagnostics and contact tracing. Continuous access to data over time was vital to understanding the pandemic’s impact on health and healthcare systems, and the effects of pharmaceutical and non-pharmaceutical interventions. Data are needed to provide inputs to calibrate, validate or fit mathematical models. Precise and accurate model inference depends on the volume, relevance and quality of available data.^47^ Therefore, improved data quality and accessibility and better information sharing are essential for models to predict and support the management of future health emergencies.^48^

A robust data infrastructure and data governance policies are necessary for building modelling ecosystems to support decision-making. This includes systems, tools and processes to collect, store, manage and analyse data, servers, data protection policies, data accessories, stable internet connectivity, GitHub accounts, hardware, cloud space and reliable electricity. Inadequate data infrastructure can compromise the accuracy and reliability of mathematical models by introducing incomplete, inaccurate or inconsistent assumptions, leading to unreliable predictions and poorly informed decisions. Additionally, sharing data and models among different organisations and researchers enables collaboration, which is crucial for the early identification of potential outbreaks and evaluating effective public health interventions. Researchers in LMICs reported challenges in access to hardware and software, restricted data access, limited storage capacity, inadequate coverage and low internet bandwidth, hindering their ability to leverage data effectively for modelling and informing policy.^44^

**Figure 4:**
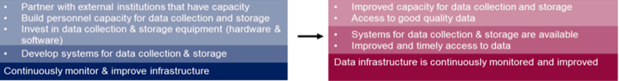
Strategies to improve data infrastructure and desired outcomes.^41^

As shown in the framework (figure 4^41^), countries can begin improving data infrastructure by working with institutions already collecting data on health and demographic indicators to improve the reliability and quality of existing data sources and infrastructure. Developing data management systems and processes simultaneously, preferably led centrally by local governments, will be essential. This may involve training in data entry, cleaning and analysis and implementing robust data governance policies to ensure data security and privacy.

In the long term, countries can improve their data management systems and tools, incorporating technologies such as electronic health records and data warehouses to improve data collection, storage and analysis efficiency and quality. Implementation of these activities should be guided by a comprehensive national data strategy that clearly outlines the goals and objectives for data collection, management and use. The approach should be aligned with national health and development priorities to improve decision-making in public health and increase accountability. Over time, these strategies can lead to establishing a sustainable data governance and management system, including dedicated staff and funding, to ensure continued data availability and quality.

Worldwide data sharing, data science methods and fast network technologies may lead to improved policy-facing modelling and more effective and timely responses to emerging disease outbreaks.^48^ There is a need for capacity building in health information systems before a pandemic occurs, allowing data linkage and room for innovation to respond appropriately in times of crisis while safeguarding personal privacy and social security.^48 49^

#### Knowledge translation

Effective communication of modelling estimates relies on conveying insights clearly and transparently to relevant stakeholders. This involves presenting results in scientific and rigorous language yet ensuring clarity and accessibility for non-expert audiences. The key to successful communication for policy entails emphasising evidence generation that informs decision-making.^47^

Effective knowledge translation necessitates the clear and concise packaging of modelling results and engagement with decision-makers to comprehend their needs and priorities.^7 50^ Mastering the art of using more straightforward language on platforms primarily used by policymakers, considering the timing and visualisation, is critical in effective science communication.^51^ This process is crucial for identifying the most appropriate communication methods, applying research findings and bridging the gap between mathematical modelling and public health policymaking.^7^ Furthermore, knowledge translation is a continuous process that requires collaboration between researchers, policymakers and implementers. The process must be iterative and adaptable to ensure the research remains relevant and applicable within the policymaking process and is tailored to the context and audience.

**Figure 5:**
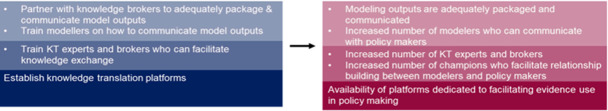
Strategies to improve knowledge translation (KT) and desired outcomes.^41^

As illustrated in figure 5,^41^ to improve capacity for knowledge translation in the short term, policymakers and researchers can engage with knowledge brokers or evidence translation experts.^52^ Knowledge brokers play a role in synthesising evidence from various sources to present a more summarised picture to policymakers, for example, by drafting policy briefs of existing research. Where brokers are unavailable, modellers can undergo training on knowledge translation and effective communication with policymakers. Developing knowledge translation platforms will be necessary in the long term to institutionalise evidence use, including mathematical models. These platforms will promote the systematic and transparent use of evidence in policymaking by providing the systems and structures, for instance, capacity, resources, infrastructure, processes, leadership and so on, needed to support evidence-informed policymaking.

#### Culture of evidence use

Studies have shown that one of the many barriers to using research evidence is the culture in which policymakers work.^53 54^ Promoting a research culture or the value placed on using research evidence in decision-making is crucial to the routine use of model estimates in policy decisions. This begins with ensuring that policymakers have access to and value models as sources of evidence by regularly interacting with researchers and other experts. The pandemic provided a policy window in which policy actors became more receptive to the use of evidence to inform their decisions, which is a first step towards embracing the culture of evidence use.^35^ The demand for evidence, however, far outweighed the capacity of many LMIC researchers to respond.^44^

**Figure 6:**
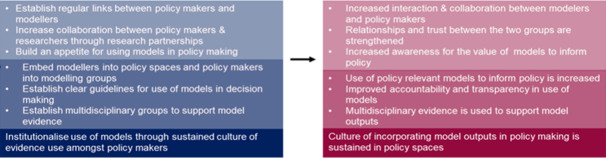
Strategies for improved culture of evidence use and desired outcomes.^41^

It is essential to leverage this momentum towards a sustained culture of evidence utilisation. As shown in figure 6,^41^ a necessary facilitator of this is improving interactions between modellers, knowledge translators, policymakers and other stakeholders, both in quality and quantity. This can be achieved by establishing regular links through stakeholder engagement activities and increasing collaborations through research partnerships, ultimately building relationships and growing trust. For example, discussions about priority questions before decision deadlines can allow modellers to create timely and valuable evidence for the policymaker, increasing trust and engagement. Research partnerships between modellers and policymakers are critical as they ensure that research is relevant to policy needs and that policy issues contribute to research agendas.

As the appetite for models increases, it can be maintained by implementing activities such as secondments that embed modellers into policy spaces and policymakers into research spaces. Such spaces should include stakeholders with multidisciplinary expertise, as model estimates are just one type of evidence within the evidence ecosystem and should be considered a piece of the puzzle. Developing transparent processes and guidelines to inform the ethical and responsible use of model estimates will be important in increasing transparency and accountability for decision-making in public health.

### Implementation approach

The improved use of model estimates in public health decision-making requires political will, continuous capacity building, applied policy-facing modelling activities and sustainable investments. Each country should set its own goals and adopt strategies for achieving them. Efforts can be prioritised according to the five components identified in this framework, with strategies considering the local context, including existing barriers and capacities. Therefore, the first step of implementation is assessing the current needs and capacity through a situational analysis and prioritising strategies based on findings. We propose a country-led implementation approach, with initiatives primarily driven by the individual governments and local implementing partners with external support when necessary, to ensure that efforts are aligned with local priorities, as exemplified by the CoMo consortium approach.^45^

Strategies to enhance the use of modelling evidence in decision-making should be participatory and developed in consultation with local stakeholders to foster buy-in and promote accountability. To ensure the impact on health outcomes and population well-being, strategies should be results-oriented, monitoring progress through tracking specific, measurable indicators, allowing continuous assessment and improvement. Finally, the strategies should be sustainably funded to ensure that results lead to long-term use of dependable, timely and locally generated modelling evidence to inform public health decisions for better health outcomes.

### Role of stakeholders

Various stakeholders must be involved in implementing the framework, and each country needs to conduct a stakeholder mapping exercise to identify the key players in public health decision-making. Here is an overview of the roles these key stakeholders could play in implementing the framework.

#### Governments

Governments and policy actors at national and subnational levels have a central role in ensuring the use of evidence to support public health decision-making. They can use the framework to assess their country’s capacity for policy-facing modelling, set sound strategies to promote the culture of evidence use and allocate adequate funding to facilitate the implementation.

#### Academic, training, modelling units and research institutions

These institutions could be responsible for providing training to develop modelling capacity and guidelines and reporting standards for modelling for policy, promoting accountability and transparency. They could also offer networks where modellers can learn from each other and get mentorship while coordinating modelling analyses and advancing methods and practices to inform policy decisions better. Teamwork would foster collaboration and avoid duplicity of functions.

#### Funding bodies

Funders could provide support to incentivise local researchers to develop skills in policy-facing mathematical modelling and encourage collaboration between different stakeholders through research partnerships. They could also support initiatives promoting evidence use during decision-making and facilitate knowledge sharing with public health professionals and policymakers, such as research findings, data sets and software tools. This can help build capacity and improve the use of modelling in these countries as governments move to provide local funding for modelling and evidence synthesis to improve the routine use of evidence for decision-making. For sustainability, we propose that government agencies offer at least some co-funding for modelling efforts to ensure buy-in.

#### Development partners

Partners could provide technical expertise about different approaches to scaling up modelling efforts for policymaking by sharing knowledge and resources with public health professionals and policymakers. They could raise awareness of models' value and utility and advocate for sustainable funding for generating and using modelled evidence in routine public health decision-making.

Further roles for development partners include convening stakeholders to develop and disseminate guidelines for decision-makers and modellers to use and apply models effectively to inform public health decisions. They can also develop and support initiatives to increase capacity for modelling, data infrastructure, knowledge translation, and evidence use.

### Monitoring framework implementation

As the implementation of this framework is highly context-specific, we cannot provide universal indicators to measure progress in the routine use of models in public health decision-making. Monitoring the outcomes of the activities proposed in red in the framework (figure 1^41^) could be used as proxy indicators, for example, dedicated government funding for policy modelling efforts, number of short courses on policy modelling, presence of modelling CoE, systems in place for data collection and storage, platforms dedicated to facilitating evidence use for policymaking and evidence of policy decisions guided by model outputs.

The report from R4D on the translation of modelled evidence for decision-making also has some indicators suggested for assessing model-related grants that could be adapted to determine the appropriate use of model outputs to inform policy decisions, for example, engagement plans in place to ensure models respond to salient policy questions; a plan to ensure data sources are clear and transparently documented; and an orientation workshop or training provided to decision-makers to enhance their understanding of modelling and their engagement in the modelling process.^43^

However, more research will be needed to determine the indicators and appropriate metrics. In the meantime, each country will have to set goals and targets that align with its capacities and commitment, allowing for the establishment of realistic and meaningful objectives. The framework’s flexibility to adapt goals, targets and measurement approaches according to each country’s unique circumstances is a strength.

### Comparison with other frameworks

Multiple frameworks for research-policy translation have been proposed, but most remain more theoretical than applied. For example, while there is consensus among scholars that building meaningful relationships between researchers and policymakers is a crucial facilitator of research-policy translation, the extant literature is largely silent on how, specifically, this can be achieved.^55^,^57^ A notable exception is Lomas’ linkage-and-exchange model,^58^ a knowledge broker model that lays out concrete steps that research foundations—conceptualised as organisations whose missions include sponsoring and disseminating research—can take to bridge the research-policy gap. One key aspect of this approach involves engaging policymakers in identifying research priorities that the foundation will support and fund. This collaborative approach ensures alignment between research agendas and the needs of policymakers, facilitating the translation of research findings into actionable policies and practices.

It is important to note that our tool is not a decision-making framework; several of these tools already exist.^59^,^61^ Rather, it is a guiding framework for building capacity for evidence-based policy decision-making by considering the critical components needed to facilitate the process. Online supplemental file 1 shows a simplified roadmap for policymakers.

## Conclusion

Mathematical modelling does not address all policy questions; modelling is only one part of a larger evidence ecosystem. There is, therefore, a need to communicate effectively to policymakers what models can and cannot do and the assumptions and limitations around models. There is also a need to encourage interdisciplinary and multisectoral collaboration for effective policy decision-making. Policymakers must make sense of the situation and link it to emerging evidence, considering the context’s constraining and enabling factors and their individual biases before making policy decisions.

We have presented a framework derived from empirical research we conducted with researchers and policy actors mainly drawn from Africa, Southeast Asia and Latin America to understand better how modelling data were used for decision-making during the pandemic, the challenges faced and what needs to be in place for future emergencies.^41^ We found that effective use of modelled evidence requires capacity building for policy-facing modelling, robust data infrastructure, sustained funding and dedicated knowledge translation mechanisms. Strong researcher–policymaker relationships and co-creation facilitated knowledge translation, while scepticism, political pressures and demand for quick outputs posed as barriers. The framework has five interdependent components aiming to guide stakeholders in lower-resourced settings about the key components to promote the use of models and the broader evidence ecosystem to inform public health policymaking. Further work is needed to implement and evaluate this framework in diverse settings to validate its robustness and determine its impact.

## supplementary material

10.1136/bmjopen-2024-093645 online supplemental file 1
