# Supplementary figures and images for: Framework to guide the use of mathematical modelling in evidence-based policy decision-making
Source: BMJ Open. 2025 Apr 5;15(4):e093645. doi: 10.1136/bmjopen-2024-093645 (PMC11973756; doi:10.1136/bmjopen-2024-093645)

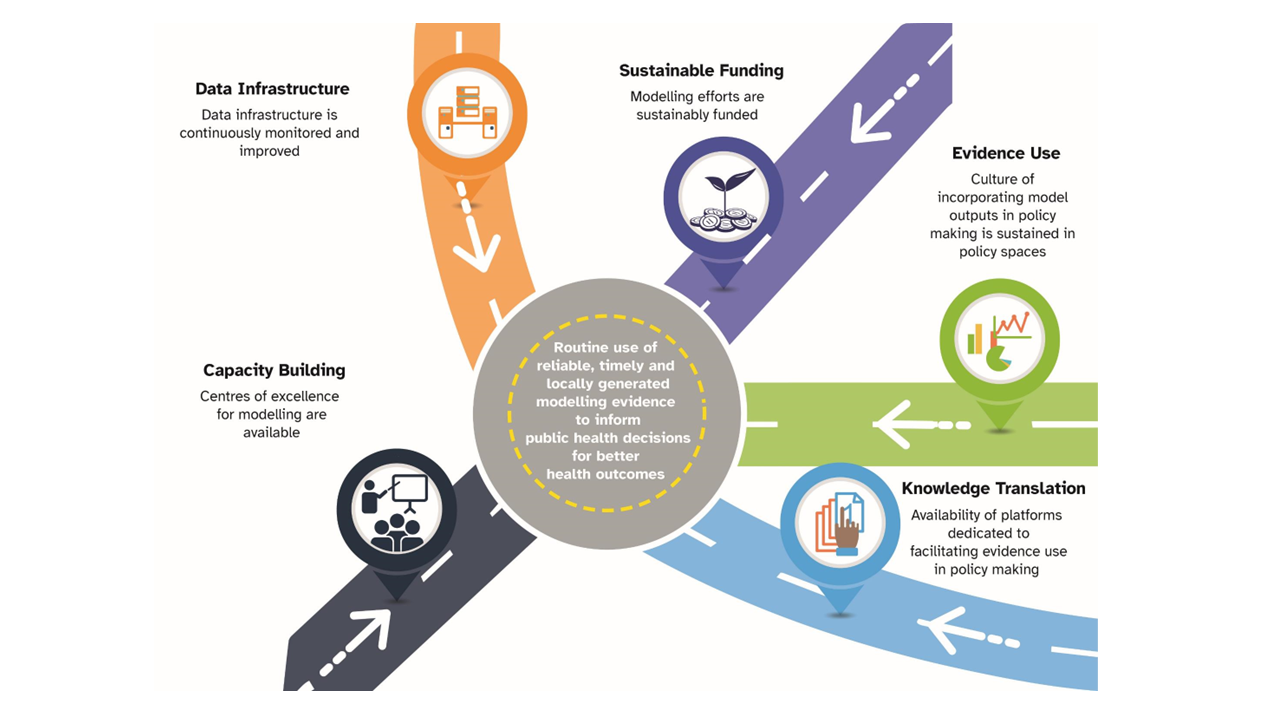

Supplement: online supplemental file 1 [file bmjopen-15-4-s001.tiff]
