# Supplementary material for: Framework to guide the use of mathematical modelling in evidence-based policy decision-making
Source: BMJ Open. 2025 Apr 5;15(4):e093645. doi: 10.1136/bmjopen-2024-093645 (PMC11973756; doi:10.1136/bmjopen-2024-093645)
Supplement: Supplementary file 2 [file bmjopen-2024-093645.reviewer_comments.pdf]

PEER REVIEW HISTORY

BMJ Open publishes all reviews undertaken for accepted manuscripts. Reviewers are asked to complete a checklist review form and are provided with free text boxes to elaborate on their assessment. These free text comments are reproduced below.

This paper was submitted to another journal from BMJ but declined for publication following peer review. The authors addressed the reviewers' comments and submitted the revised paper to BMJ Open. The paper was subsequently accepted for publication at BMJ Open.

ARTICLE DETAILS

Title (Provisional)

A FRAMEWORK TO GUIDE THE USE OF MATHEMATICAL MODELLING IN EVIDENCE-BASED POLICY DECISION-MAKING

Authors

Oliwa, Jacquie; Guleid, Fatuma Hassan; Owek, Collins J; Maluni, Justinah; Jepkosgei, Juliet; Nzinga, Jacinta; Were, Vincent; Sim, So Yoon; Walekhwa, Abel W; Clapham, Hannah; Dabak, Saudamini; KC, Sarin; Hadley, Liza; Undurraga, Eduardo; Hagedorn, Brittany; Hutubessy, Raymond

---

VERSION 1 - REVIEW

**Reviewer**	**1**
**Name**	**Reviewer 1**
**Affiliation**	
**Date**	**13-Aug-2024**
**COI**	**No competing interest**

Comments not published pending reviewer's consent.

**Reviewer**	**2**
**Name**	**Reviewer 2**
**Affiliation**	
**Date**	**13-Aug-2024**
**COI**	**No competing interest**

Comments not published pending reviewer's consent.

VERSION 2 - REVIEW

**Reviewer**	**1**
**Name**	**Vyklyuk, Yaroslav**
**Affiliation**	**Lviv Polytechnic National University, The Department of Artificial Intelligence**
**Date**	**18-Nov-2024**
**COI**	**None**

Strengths of the Article

Timely Focus: The emphasis on the importance of modelling during the COVID-19 pandemic is relevant and highlights an under-addressed issue in lower-resource settings.

Contextual Relevance: The framework appropriately identifies challenges faced by LMICs, including limited funding, capacity, and data infrastructure.

Comprehensive Scope: The article touches on a wide range of factors influencing the adoption of mathematical models, such as funding, knowledge translation, and data infrastructure.

Lack of Originality in Contribution:

1. The article fails to provide quantitative analysis or comparative metrics to validate its framework against existing methods. This limits its contribution to the body of research. While it claims to address gaps in modelling for low- and middle-income countries, the lack of empirical evaluation of other frameworks renders its recommendations subjective and potentially biased.

2. Absence of Concrete Recommendations:

The paper presents general ideas and concepts rather than actionable steps. No quantifiable financial implications, cost estimates, or resource requirements for implementing the proposed framework are discussed. Moreover, there is no evaluation of the tools or frameworks currently in use in mathematical modelling for public health.

3. Superficial Treatment of Mathematical Modelling:

While the framework mentions mathematical modelling broadly, it lacks a technical or detailed analysis of specific mathematical approaches, such as reinforcement learning or simulation-based modelling. This weakens its appeal to a technically knowledgeable audience who would expect in-depth examination and validation of models.

4. Need for Quantitative Comparison and Validation:

A quantitative comparison of different approaches or factors mentioned in the paper is necessary to validate the framework's robustness. Additionally, providing a detailed representation of the proposed framework and supporting it with real-world examples or case studies would greatly enhance its credibility.

5. Philosophical Over Practical Approach:

The narrative style of the article leans heavily on theoretical discussions and philosophical reflections about modelling rather than evidence-backed or practically implementable guidance. This detracts from its utility as a decision-making tool for policymakers or public health practitioners.

Suggestions for Improvement

1. Introduce Comparative Metrics:

Incorporate a quantitative evaluation of the proposed framework against existing frameworks. Metrics such as efficiency, cost-effectiveness, and outcomes in specific public health scenarios should be included.

2. Develop Actionable Steps:

Provide specific recommendations, such as a roadmap for building capacity or a stepwise guide for implementing the framework. Include details on financial costs and resource requirements.

3. Include Technical Depth:

Expand the discussion of mathematical models by including specific methods and their potential applications. For instance, detail the role of simulation models, neural networks, or reinforcement learning in policy decision-making.

4. Validate the Framework with Case Studies:

Present real-world applications or pilot implementations of the framework in LMICs, supported by empirical data.

5. Strengthen Visual and Quantitative Representations:

Include detailed visualizations of the framework and provide quantitative data or simulations to support the claims.

Final Recommendation

The article has potential but falls short in methodological rigor and actionable outcomes. A major revision is recommended to address the issues outlined above, ensuring the framework offers tangible value to the target audience and aligns with evidence-based academic standards.

Reviewer 2

**Name**	**Champagne, Clara**
**Affiliation**	**Swiss Tropical and Public Health Institute**
**Date**	**03-Dec-2024**
**COI**	**None**

In this manuscript, Oliwa and colleagues propose ideas to guide how to foster the use of disease mathematical modelling for decision-making. These ideas are organized into a framework targeted at global stakeholders (policy-makers, academics, funders, implementing partners), especially in lower-resourced settings. The main areas highlighted for the framework are related to funding, capacity strengthening, data infrastructure, knowledge translation and climate of evidence use. This framework was derived through a methodology presented in a separated article: https://gh.bmj.com/content/9/11/e015247. The conclusions were also outlined in a policy brief (pre-print equivalent?): https://kemri-wellcome.org/framework-to-guide-the-use-of-mathematical-modelling-in-evidence-based-policy-decision-making/.

I think the article tackles a very important topic and the ideas are useful and relevant for the community. In line with the journal's requirements, it emphasizes the roles of collaboration, transparency and openness among various stakeholders for more evidence use in decision-making on disease control.

However, I think the current manuscript would benefit from several major improvements in order to be suitable for publication, as detailed below.

1. Highlight key recommendations from the framework:

The abstract and summary explain more the methodology and the purpose of the framework but reflect very little from its content, apart from listing the 5 dimensions (funding, capacity strengthening, data infrastructure, knowledge translation and climate of evidence use). I believe it would be helpful to better summarize the key recommendations that emerge from the framework, especially in the abstract, the summary and the conclusion.

2. Better clarify the difference and added-value with companion paper:

The authors mention clearly that the methodology is presented in a companion paper and that the current work presents a framework derived from it. It would nonetheless be helpful to better highlight what were the main learnings from the companion paper and what the current manuscript brings as additional information / knowledge / perspective.

3. Figures

Figure 1 is identical to the Figure 5 in the companion paper (https://gh.bmj.com/content/9/11/e015247). Figures 2 to 6 are repeated subsets of Figure 1. The very last figure is also a simplified version of Figure 1 (and it is present in the policy

brief). Additionally, Figures 1 to 6 are in low resolution and pixelated, making them hard to read. I would encourage the authors to rethink the figures to be included in this article in order to reduce repetition, duplication and improve reading quality.

4. Scope in terms of disease areas

The introduction part makes an effort to define mathematical modelling, but I think it would be useful to expand this section a little, to be very specific about 1/what is meant with disease modelling, 2/what disease areas are considered (infectious, emergent, any, etc.) 3/ what type of decision-making is referred to in the manuscript.

5. Additional comment: to the best of my knowledge, the Pasteur institute in France is an independent (research) institution and not a government agency, so the text line 117 should be edited to reflect this.

**Reviewer**	**3**
**Name**	**Yang, Junyuan**
**Affiliation**	**Complex Systems Research Center, Shanxi University**
**Date**	**04-Dec-2024**
**COI**	**None**

I am happy with the authors' response

VERSION 2 - AUTHOR RESPONSE

Reviewer: 1

Strengths of the Article

Timely Focus: The emphasis on the importance of modelling during the COVID-19 pandemic is relevant and highlights an under-addressed issue in lower-resource settings.

Contextual Relevance: The framework appropriately identifies challenges faced by LMICs, including limited funding, capacity, and data infrastructure.

Comprehensive Scope: The article touches on a wide range of factors influencing the adoption of mathematical models, such as funding, knowledge translation, and data infrastructure.

Thank you for your comments on the strengths of our article.

Lack of Originality in Contribution:

1. The article fails to provide quantitative analysis or comparative metrics to validate its framework against existing methods. This limits its contribution to the body of research. While it

claims to address gaps in modelling for low- and middle-income countries, the lack of empirical evaluation of other frameworks renders its recommendations subjective and potentially biased.

Thank you. The framework was recently co-created with researchers and policymakers from nearly 40 global south countries through a rigorous process. While we acknowledge the comment about the lack of validation, it is a new tool, in early phases of use, and still adds value as an evidence-based guiding framework to increase capacity for use of modelling for policy decision-making in lower resourced settings. Early feedback from stakeholders that have reported its utility, and we have been invited to support implementation in various settings. Our aim for presenting it here is to increase awareness of its availability, and perhaps empirical studies can follow in the future to evaluate its use.

2. Absence of Concrete Recommendations:
The paper presents general ideas and concepts rather than actionable steps. No quantifiable financial implications, cost estimates, or resource requirements for implementing the proposed framework are discussed. Moreover, there is no evaluation of the tools or frameworks currently in use in mathematical modelling for public health.

The framework does provide actionable steps (see Fig 1, the blue column on proposed activities based on one's assessment score), and the implementation section (lines 387-467)

We are not able to provide quantifiable financial implications, cost estimates, or resource requirements as these would require data from process and impact evaluations, which we do not have at this stage.

We compared tools/frameworks currently in use on lines 468-485 but did not analyse or evaluate them as that was outside the scope of what was intended for this communication piece, which must be succinct. Further comparative work would need empiric research, which we have added to the recommendations (lines 505-506).

3. Superficial Treatment of Mathematical Modelling:
While the framework mentions mathematical modelling broadly, it lacks a technical or detailed analysis of specific mathematical approaches, such as reinforcement learning or simulation-based modelling. This weakens its appeal to a technically knowledgeable audience who would expect in-depth examination and validation of models.

Technical/detailed analysis of mathematical approaches was outside the scope of this communication piece, which targeted a broader/lay audience on knowledge translation for modelling evidence in non-technical jargon. Modellers were involved in co-creating the framework, and they have found it useful to engage with their policymakers to increase the demand for applied modelling in policy decision-making. We did not intend to do an in-depth examination and validation of models for this work.

4. Need for Quantitative Comparison and Validation:
A quantitative comparison of different approaches or factors mentioned in the paper is necessary to validate the framework's robustness. Additionally, providing a detailed representation of the proposed framework and supporting it with real-world examples or case studies would greatly enhance its credibility.

This would be the next step, implementation and evaluation of the framework (added to recommendations). This communication piece was intended to increase awareness of its availability and thus invite users to implement it in different settings and evaluate its robustness.

5. Philosophical Over Practical Approach:
The narrative style of the article leans heavily on theoretical discussions and philosophical reflections about modelling rather than evidence-backed or practically implementable guidance. This detracts from its utility as a decision-making tool for policymakers or public health practitioners.

The narrative style was chosen for a communication article. The framework was derived from a review of the literature and empirical research we conducted with researchers and policy actors mainly drawn from Africa, Southeast Asia, and Latin America, making it evidence-based. We provide actionable steps in the proposed activities, as well as metrics to measure success which will aid in implementation as illustrated in Figure 1 and in subsequent sections. We have also received feedback from various stakeholders (early adopters) who have found the tool useful, and thus, we have decided to now make it widely available through the BMJ Open readership.

Suggestions for Improvement

1. Introduce Comparative Metrics:
Incorporate a quantitative evaluation of the proposed framework against existing frameworks. Metrics such as efficiency, cost-effectiveness, and outcomes in specific public health scenarios should be included.

Thank you for the suggestion, but this goes outside the intended scope of the communication piece, and can be added as a recommendation for future work.

2. Develop Actionable Steps:
Provide specific recommendations, such as a roadmap for building capacity or a stepwise guide for implementing the framework. Include details on financial costs and resource requirements.

The framework (see blue column in Fig 1) provides specific actions that each country can take as it implements it. Financial costs and resource requirements will be context-specific and require local experts to project. We do not have any costing data at this point.

3. Include Technical Depth:
Expand the discussion of mathematical models by including specific methods and their potential applications. For instance, detail the role of simulation models, neural networks, or reinforcement learning in policy decision-making.

Thank you for the suggestion, but our research did not study simulation models, neural networks or reinforcement learning as these were not part of our objectives and such can not feature in this communication piece.

4. Validate the Framework with Case Studies:
Present real-world applications or pilot implementations of the framework in LMICs, supported by empirical data.

We have included this in recommendations for future work.

5. Strengthen Visual and Quantitative Representations:
Include detailed visualizations of the framework and provide quantitative data or simulations to support the claims.

The figures we have provided was our attempt at providing detailed visualizations of the framework, we do not have quantitative data or simulations unfortunately.

Final Recommendation
The article has potential but falls short in methodological rigor and actionable outcomes. A major revision is recommended to address the issues outlined above, ensuring the framework offers tangible value to the target audience and aligns with evidence-based academic standards.

Thank you for taking time to review and your feedback.

Reviewer: 2

In this manuscript, Oliwa and colleagues propose ideas to guide how to foster the use of disease mathematical modelling for decision-making. These ideas are organized into a framework targeted at global stakeholders (policy-makers, academics, funders, implementing partners), especially in lower-resourced settings. The main areas highlighted for the framework are related to funding, capacity strengthening, data infrastructure, knowledge translation and climate of evidence use. This framework was derived through a methodology presented in a separated article: https://gh.bmj.com/content/9/11/e015247. The conclusions were also outlined in a policy brief (pre-print equivalent?): https://kemri-wellcome.org/framework-to-guide-the-use-of-mathematical-modelling-in-evidence-based-policy-decision-making/.

I think the article tackles a very important topic and the ideas are useful and relevant for the community. In line with the journal's requirements, it emphasizes the roles of collaboration, transparency and openness among various stakeholders for more evidence use in decision-making on disease control.

Thank you for taking the time to review our article and for your feedback.

However, I think the current manuscript would benefit from several major improvements in order to be suitable for publication, as detailed below.

1. Highlight key recommendations from the framework:
The abstract and summary explain more the methodology and the purpose of the framework but reflect very little from its content, apart from listing the 5 dimensions (funding, capacity strengthening, data infrastructure, knowledge translation and climate of evidence use). I believe it would be helpful to better summarize the key recommendations that emerge from the framework, especially in the abstract, the summary and the conclusion.

Noted with thanks, we have now included a key recommendation: implement and evaluate the framework in different settings to generate evidence of its robustness and potential impact (lines 505-506, and in the abstract lines 66-67 and summary box)

2. Better clarify the difference and added-value with companion paper:
The authors mention clearly that the methodology is presented in a companion paper and that the current work presents a framework derived from it. It would nonetheless be helpful to better highlight what the main learnings from the companion paper were and what the current manuscript brings as additional information / knowledge / perspective.

Noted with thanks, the companion paper describes the process of collecting the empirical data and the findings, which contributed to the development of the framework, which was only briefly

introduced in the companion paper. We have included a brief summary of the lessons learned, linking to the framework development in the abstract (lines 44-48), introduction (lines 144-148) and conclusion (lines 498-502).

This communication piece goes into detail about each of the components of the framework, giving the evidence behind each component and suggestions for how it can be implemented.

3. Figures
Figure 1 is identical to the Figure 5 in the companion paper (https://gh.bmj.com/content/9/11/e015247). Figures 2 to 6 are repeated subsets of Figure 1. The very last figure is also a simplified version of Figure 1 (and it is present in the policy brief). Additionally, Figures 1 to 6 are in low resolution and pixelated, making them hard to read. I would encourage the authors to rethink the figures to be included in this article in order to reduce repetition, duplication and improve reading quality.

Thank you. Indeed, Figure 1 and Figure 5 are identical because we only briefly introduced this framework in the companion paper, and now in this paper, we plan to go into greater detail about its components and how best to implement it. The subset images are indeed derived from the main framework, and we use them as is to elucidate the different components of the framework.

We did attempt to adjust the resolution to the journal's dpi requirements, and hopefully after editing they will be clearer.

4. Scope in terms of disease areas
The introduction part makes an effort to define mathematical modelling, but I think it would be useful to expand this section a little, to be very specific about 1/what is meant with disease modelling, 2/what disease areas are considered (infectious, emergent, any, etc.) 3/ what type of decision-making is referred to in the manuscript.

Noted, the framework is designed to be generic and disease-agnostic for any policy decision-making to which modelling could apply. Policy-facing modelling (i.e., creating and using mathematical models tailored to inform policy decisions in public health), so whether for vertical immunisation programmes or pandemics, different models can be developed to guide decision-making. The framework is thus more about encouraging knowledge translation, and the decisions will depend on the questions asked and whether they are amenable to modelling. We had given some examples of questions policymakers grappled with during the pandemic (lines 81-83), which could have been partly answered by modelling, e.g. how many people would become infected, how far it could spread, which public health interventions would be effective, how resources should be optimally allocated, etc.

5. Additional comment: to the best of my knowledge, the Pasteur institute in France is an independent (research) institution and not a government agency, so the text line 117 should be edited to reflect this.

Noted with thanks, we included it as one of the institutions that are actively involved in policy-facing modelling, not as a government agency, and have edited the line 117 to reflect this.

Reviewer: 3
Dr. Junyuan Yang, Complex Systems Research Center, Shanxi University
Comments to the Author:

I am happy with the authors' response

VERSION 3 - REVIEW

**Reviewer**	**2**
**Name**	**Champagne, Clara**
**Affiliation**	**Swiss Tropical and Public Health Institute**
**Date**	**07-Feb-2025**
**COI**	

I thank the authors for their answers and their revised manuscript. I don’t have any further remark on the overall message of this communication article, which I believe addresses an important and relevant topic.

However, I think that my remarks on the formal aspects have not been properly addressed.

I don’t know what the rules are for communication articles as opposed to original scientific articles, but the figures in the current manuscript are still identical to a previous publication, which at the very least, should be properly cited in their legend (if not removed or replaced by the supplementary figure).

I also believe that “mathematical modeling” could be better defined, as it is an term sometimes used to refer to a very large variety of approaches, including machine learning, dynamical systems or statistical methods among others. Regarding the scope in disease areas, I thank the authors for their response and believe that some elements provided in their response could be included in the manuscript.

The added value of the current manuscript compared to the previous article is better detailed but the abstract is now a little repetitive, so potential reformulations would be welcome.

VERSION 3 - AUTHOR RESPONSE

Response to Reviewer 2

I thank the authors for their answers and their revised manuscript. I don’t have any further remark on the overall message of this communication article, which I believe addresses an important and relevant topic.

However, I think that my remarks on the formal aspects have not been properly addressed.
I don’t know what the rules are for communication articles as opposed to original scientific

articles, but the figures in the current manuscript are still identical to a previous publication, which at the very least, should be properly cited in their legend (if not removed or replaced by the supplementary figure).

Noted with thanks, we have now added citation 41 to all the figures, their legends, and within the text, linking to the companion paper where the framework is first illustrated. We needed the broken-down figures to illustrate the various components of the framework separately; the supplementary figure then shows how they are interlinked to support the end goal, and thus opted not to remove them.

I also believe that “mathematical modeling” could be better defined, as it is an term sometimes used to refer to a very large variety of approaches, including machine learning, dynamical systems or statistical methods among others. Regarding the scope in disease areas, I thank the authors for their response and believe that some elements provided in their response could be included in the manuscript.

This has now been edited, lines 90-92, lines 103-105.

We have also added the line to clarify the scope in the abstract (lines 55-56) and in the main text 159-160).

The added value of the current manuscript compared to the previous article is better detailed but the abstract is now a little repetitive, so potential reformulations would be welcome.

Noted with thanks, the whole abstract has been edited for clarity.
